# Chylothorax and chylous ascites due to juvenile paracoccidioidomycosis: a case report

**DOI:** 10.1590/0037-8682-0605-2022

**Published:** 2023-05-22

**Authors:** Ana Paula Freitas Bahia dos Santos, Tomás Varella Costa Russo, Beatriz Pascuotte, Luis Felipe Silva Visconde, Adryelle Carolynne Nogueira Luetz, Andrey Biff Sarris, Matheus Dias Girão Rocha, Fernanda Guioti Puga, Gilberto Gambero Gaspar, Valdes Roberto Bollela, Roberto Martinez

**Affiliations:** 1 Faculdade de Medicina de Ribeirão Preto, Hospital das Clínicas, Departamento de Clínica Médica, Infectologia, Ribeirão Preto, SP, Brasil.

**Keywords:** Paracoccidioidomycosis, Chylothorax, Chylous Ascites

## Abstract

Paracoccidioidomycosis (PCM) is a systemic fungal infection caused by *Paracoccidioides* species*.* Chylothorax is a rare complication of PCM. A 16-year-old adolescent presented daily fever, lymphadenomegaly, sweating, weight loss, ventilatory-dependent pain, and dysphagia, which confirmed PCM. During treatment, the patient developed chylothorax and chylous ascites. Chronic inflammatory and fibrotic lymphadenopathy may obstruct lymphatic vessels, resulting in the extravasation of lymph into the abdomen or pleural cavities. Chylothorax is one of several complications of PCM and can lead to respiratory insufficiency, even in patients undergoing antifungal therapy.

## INTRODUCTION

Paracoccidioidomycosis (PCM) is an endemic systemic fungal infection with an under-reported incidence in Brazil and Latin America^1^ ^,^ ^2^. The incidence of PCM is approximately 1-3 cases per 100,000 inhabitants annually in endemic regions. *Paracoccidioides brasiliensis* species complex and *Paracoccidioides lutzii* are causative agents that may cause induce damage in almost all tissues, with various clinical signs and symptoms^1^. PCM is classified into two clinical presentations, acute/subacute or juvenile and chronic or adult forms, despite the possibility of varied manifestations^1^ ^,^ ^3^.

The chronic form (adult PCM) is the most frequent presentation, with a prevalence of 74-96%. It is highly prevalent in adults between 30-60 years. Unlike the juvenile form, adult PCM has a 22:1 male-to-female incidence ratio. Its clinical course is insidious, with symptoms ranging between 4-6 months, occasionally reaching more than one year^1^.

Acute/subacute or juvenile PCM accounts for 5-25% of PCM cases. With an equal gender distribution, it is more prevalent in children and adolescents, although it might affect young adults who are seldom older than 30 years of age. In addition, juvenile PCM has a short clinical course (average, two months)^1^ ^,^ ^3^.

The acute form of the disease rapidly spreads to multiple organs and systems, primarily the mononuclear phagocytic system. Generalized lymphadenomegaly and hepatosplenomegaly are well-known primary manifestations^1^ ^,^ ^4^. Complications such as chylous ascites, chronic liver disease, and portal hypertension are uncommon in juvenile PCM^5^. Chylothorax, a manifestation of PCM, is a rare complication. To the best of our knowledge, only one such case has been documented in the literature^6^.

The present report describes a case of chylothorax in a patient with PCM. Although potentially fatal, this is considered rare. PCM is an endemic disease prevalent in most of Latin America^2^. It is necessary to highlight this condition and include it in the differential diagnosis of chylothorax. 

## CASE REPORT

A previously healthy 16-year-old male born in Alfenas, Minas Gerais, but residing in the urban area of São José do Rio Preto, São Paulo, Brazil, was admitted to a university hospital. He complained of experiencing generalized lymphadenomegaly for three weeks. He had a history of tobacco consumption (5-6 cigarettes/day), use of illicit drugs such as marijuana and cocaine, and gardening.

He also reported daily vespertine fever associated with fatigue, prostration, sweating, and a 10-kg weight loss. Furthermore, the patient complained of ventilatory-dependent pain in the right hemithorax and low dysphagia with solid food intake. Physical examination revealed that the liver was palpable, 4 cm below the right costal margin, with palpable lymph nodes, 1-3 cm in all superficial chains, mobile, and painful.

**FIGURE 1:**
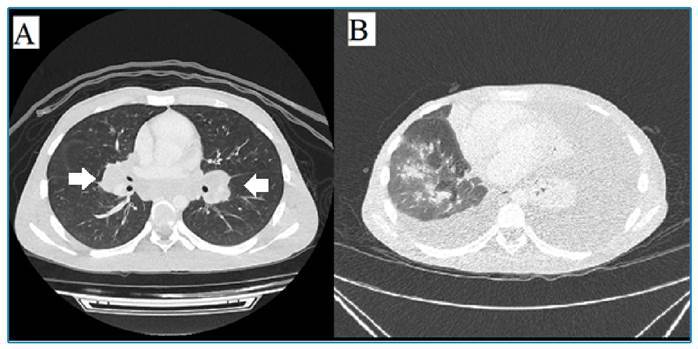
Chylotorax due to juvenile paracoccidioidomycosis.

Imaging tests performed during hospital admission revealed mesenteric, retroperitoneal, pelvic, inguinal, mediastinal, and hilar lymphadenopathy, hepatosplenomegaly, and large-volume ascites (Figure 1). Initial laboratory tests revealed normocytic and normochromic anemia; leukocytosis; neutrophilia with a left shift; intense eosinophilia; and elevated blood alkaline phosphatase, gamma-glutamyl transferase, and C-reactive protein levels. 

**Figure legend:** Thorax contrasted tomography (axial view) showing a bilaterally enlarged lymph node - arrows - **(A)** and a bilateral pleural effusion **(B).**

Chylous ascites was characterized by comparing lipid levels in these fluids to serum levels (Table 1).

**FIGURE 2:**
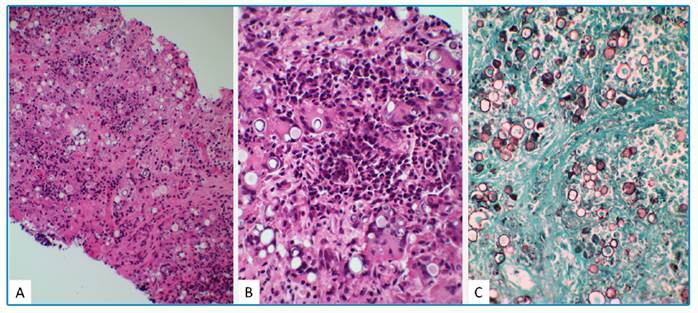
Inguinal lymph node biopsy with fungal structures suggesting *Paracoccidioides brasiliensis.*

An inguinal lymph node biopsy revealed granuloma formation with epithelioid histiocytes and multinucleated giant cells, numerous circular and oval fungal structures considerably varying in size, and some with multiple exosporulations inside and outside granulomas, suggesting *Paracoccidioides* spp. (Figure 2). 

**Figure legend:**Inguinal lymph node biopsy with granuloma formation and round fungal forms in hematoxylin-eosin (HE) stained sections 200× **(A)**. HE 400× with loose granulomas and fungal structures, with multiple sporulation, suggesting*Paracoccidioides*spp.**(B)** and 400× Grocott-Gomori methenamine-silver (GMS) stain with round fungal forms with multiple exosporulations **(C)**.

Additionally, in the counterimmunoelectrophoresis test, anti-*Paracoccidioides* spp. and anti-*Histoplasma capsulatum* serum antibodies had titers of 1:512 and 1:1, respectively. We initiated treatment with amphotericin B deoxycholate at 1 mg/kg/day, followed by liposomal amphotericin B (2 mg/kg/day) after five days. After 12 days, amphotericin B was replaced with itraconazole (400 mg/day).

During itraconazole therapy, the patient developed bilateral chylous pleural effusion (Figure 1, Table 1) with exacerbated chylous ascites. Consequently, the patient frequently undergoes therapeutic paracentesis and thoracentesis. 

**TABLE 1:** Ascitic and pleural fluid cytological and biochemical characteristics.

Lab Exam	Ascitic Fluid	Pleural Fluid
Appearance	Milky/Hemorrhagic	Serosanguineous
Red blood cells (cel.10³/ µl)	<10	38
White blood cells (/µl)	290	4 363
Lymphocytes (%)	86	-
Polymorphonuclear cells (%)	12	35.3
Mononuclear cells (%)	-	63.4
Cholesterol (mg/dL)	-	54.1
Triglycerides (mg/dL)	253.59	132.94
Lactate dehydrogenase (U/L)	34	140.8
pH	7.466	7
Total proteins (g/dL)	2	2.79
Glucose (mg/dL)	126	-

On day 33 of hospitalization, the patient presented with deteriorated ventilatory status, necessitating orotracheal intubation, resulting in a 14-day stay in the intensive care unit (ICU). During this period, intravenous sulfamethoxazole-trimethoprim (800/160 mg) was administered every 8 h. However, after eight days, the treatment was changed to liposomal amphotericin B (2 mg/kg/day). Simultaneously, octreotide (0.1 mg) was administered every 8 h to address chylous ascites, and the patient also underwent pleural drain insertion. Octreotide was administered for 16 days. 

On day 43, post-extubation, the patient developed a colonic pseudo-obstruction and was treated conservatively. On day 57, the patient was returned to the Infectious Diseases ward under low-fat oral feeding. Given the worsening chylous ascites, oral corticosteroid therapy with prednisone of 1 mg/kg/day was administered to reduce the obstructive lymph node volume, although no response was achieved. On day 59, the antimicrobial therapy was replaced with intravenous sulfamethoxazole-trimethoprim (800/160 mg) every 8 h. Subsequently, on day 64, the same dose of sulfamethoxazole-trimethoprim was administered orally.

Given the absence of low rates in thoracic drains, bilateral chemical pleurodesis was performed on day 66. After the procedure, the patient developed tachypnea and chest pain, necessitating supplemental oxygen therapy. On day 67, a new orotracheal intubation was performed. Subsequently, a tracheostomy was performed. The patient was administered antibacterial drugs during ICU admission to treat healthcare-related infections.

On day 84, the patient experienced cardiorespiratory arrest, which reversed after 10 min. Subsequently, the patient showed remarkable improvement without neurological sequelae and was successfully weaned from sedoanalgesia. 

Although the patient experienced multiple secondary bacterial infections during hospitalization, recovery was good. Eventually, the tracheostomy tube was removed, and oral feeding was restarted. Furthermore, pleural effusion did not recur after pleurodesis. Paracentesis was no longer required, and ascites resolved entirely. At the end of hospitalization, the accumulated amphotericin B dose was 6,500 mg.

## DISCUSSION

The main symptoms of acute/subacute PCM are localized or generalized lymphadenomegaly and hepatosplenomegaly. Other symptoms include fever, weight loss, anorexia, digestive manifestations (abdominal pain, palpable masses, diarrhea, jaundice, ascites, and intestinal subocclusion), and cutaneous/mucosal and osteoarticular lesions^1^ ^,^ ^2^ ^,^ ^4^.

Unlike the chronic form, pulmonary involvement is less common in juvenile PCM (70-100% compared with 10-20%, respectively)^1^ ^,^ ^4^. Thoracic radiography may reveal hilar and paratracheal lymph node enlargement, pulmonary infiltration, and rare pleural effusion|^4^ ^,^ ^7^. According to Mendes et al, lymphadenomegaly and pleural effusion can be detected in 50 and 2% of chest radiographs, respectively^3^. Imaging tests involving the abdomen showed the presence of hepatosplenomegaly, ganglionary masses, bile duct dilatation (due to extrinsic compression), and ascites^4^.

A prospective study involving 63 children with PCM identified lymph node enlargement as the most frequent clinical manifestation, with lymph node fistulization occurring in 7 cases (10%). Lymphadenomegaly is the main finding on chest radiographs, commonly in the abdomen^8^.

Some reports^1^ ^,^ ^4^ have confirmed that enlarged intra-abdominal lymph nodes may coalesce, leading to tumor masses and consequently compressing various organs. This information has led specialists to discuss the possible mechanisms involved in the development of chylous ascites or chylothorax. Lymphadenomegaly is a common finding in PCM and occurs in other conditions (including malignancy, sarcoidosis, amyloidosis, and tuberculosis). It may compress the inner lymphatic vessels, resulting in extravasation of the chyle into the abdomen or pleural cavities^6^ ^,^ ^9^ ^,^ ^10^. Moreover, lymph node inflammatory necrosis and fibrosis can result in the blockade of lymphatic circulation, dilation of lymphatic vessels, and loss of lymph in tissues and corporal cavities^11^.

A diagnosis of chylothorax and chylous ascites can be reached based on fluid analysis. The diagnostic criteria for chylothorax include pleural fluid triglyceride level > 110 mg/dL, cholesterol level < 200 mg/dL, pleural fluid-to-serum triglyceride ratio > 1, and pleural fluid-to-serum cholesterol ratio ˂1^10^. Alternatively, the diagnosis of chylous ascites is based on the milky appearance of the liquid and a triglyceride level of >200 mg/dL. In the present case report, the patient had a pleural fluid triglyceride level of 132.94 mg/dL, serum triglyceride level of 90,81 mg/dL, a pleural fluid cholesterol level of 54.10 mg/dL, serum cholesterol level of 125,95 mg/dL, a pleural fluid-to-serum triglyceride ratio of 1.46, and a pleural fluid-to-serum cholesterol ratio of 0.42. The patient values met the criteria for the presence of chylous ascites and chylothorax.

Mesenteric lymphadenopathy also causes edema of the intestinal mucosa and malabsorption of proteins and fats^1^ ^,^ ^3^ ^,^ ^12^. Loss of immunoglobulin and lymphocytes frequently accompany protein loss in these patients, which can lead to a deficiency in humoral- and cell-mediated immunity^12^. Considering the present patient, the loss of lymph, rich in proteins and lymphocytes, might have contributed to the poor therapeutic response to oral antifungals and led to immunosuppression, facilitating the occurrence of secondary infections requiring intensive care.

In conclusion, this report describes a case of chylous ascites and chylothorax, which are rare and potentially fatal complications of the acute/subacute or juvenile form. The inclusion of PCM in the differential diagnosis of chylothorax and ascites is necessary, especially in endemic areas such as South America.
